# KPr(PO_3_)_4_

**DOI:** 10.1107/S1600536810026942

**Published:** 2010-07-14

**Authors:** Abdelghani Oudahmane, Mohamed Daoud, Boumediene Tanouti, Daniel Avignant, Daniel Zambon

**Affiliations:** a Université Cadi Ayyad, Laboratoire de la Matière Condensée et de l’Environnement, Faculté des Sciences Semlalia, Département de Chimie, BP 2390, 40000 Marrakech, Morocco; b Université Blaise Pascal, Laboratoire des Matériaux Inorganiques, UMR CNRS 6002, 24 Avenue des Landais, 63177 Aubière, France

## Abstract

Single crystals of the title compound, potassium praseodymium(III) polyphosphate, were obtained by solid-state reaction. The monoclinic non-centrosymmetric structure is isotypic with all other K*Ln*(PO_3_)_4_ analogues from *Ln* = La to Er, inclusive. The crystal structure of these long-chain polyphosphates is built up from infinite crenelated polyphosphate chains of corner-sharing PO_4_ tetra­hedra with a repeating unit of four tetra­hedra. These chains, running along [100], are arranged in a pseudo-tetra­gonal rod packing and are further linked by isolated PrO_8_ square anti­prisms [Pr—O = 2.3787 (9)–2.5091 (8) Å], forming a three-dimensional framework. The K^+^ ions reside in channels parallel to [010] and exhibit a highly distorted coordination sphere by eight O atoms at distances ranging from 2.7908 (9) to 3.1924 (11) Å.

## Related literature

Long-chain polyphosphates with general formula *A* ^I^ *B* ^III^(PO_3_)_4_ have been classified into seven structural types, labelled from I to VII (Jaoudi *et al.*, 2003 ▶). All K*Ln*(PO_3_)_4_ polyphosphates (*Ln* is a trivalent rare earth element) reported up to now adopt type III except for KYb(PO_3_)_4_ (Palkina *et al.*, 1981 ▶). For corresponding isotypic crystal structures, see: Zhu *et al.* (2009 ▶) for Ce and Eu; Horchani-Naifer *et al.* (2008 ▶) for Y; Parreu *et al.* (2006 ▶) for Gd and Nd; Xing *et al.* (1987 ▶) for Tb; Ninghai *et al.* (1984 ▶) for Eu; Lin *et al.* (1983 ▶) for La; Krutik *et al.* (1980 ▶) for Er; Hong *et al.* (1975 ▶) for Nd. For a review of the crystal chemistry of phosphates, see: Durif (1995 ▶). For the cyclo­phosphate structure with the same composition, KPr(PO_3_)_4_, see: Zhou *et al.* (1987 ▶).

## Experimental

### 

#### Crystal data

KPr(PO3)_4_
*M* *_r_* = 495.89
Monoclinic, 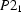

*a* = 7.2872 (2) Å
*b* = 8.4570 (3) Å
*c* = 8.0268 (2) Å
β = 91.994 (1)°
*V* = 494.37 (3) Å^3^
*Z* = 2
Mo *K*α radiation
μ = 6.06 mm^−1^
*T* = 296 K
0.29 × 0.21 × 0.16 mm

#### Data collection

Bruker APEXII CCD diffractometer
Absorption correction: multi-scan (*SADABS*; Bruker, 2008 ▶) *T*_min_ = 0.448, *T*_max_ = 0.751
45940 measured reflections
13443 independent reflections
13257 reflections with *I* > 2σ(*I*)
*R*_int_ = 0.034

#### Refinement

*R*[*F* ^2^ > 2σ(*F* ^2^)] = 0.018
*wR*(*F* ^2^) = 0.043
*S* = 1.07
13443 reflections
164 parameters
1 restraint
Δρ_max_ = 2.86 e Å^−3^
Δρ_min_ = −2.42 e Å^−3^
Absolute structure: Flack (1983 ▶), 6278 Friedel pairs
Flack parameter: 0.022 (3)

### 

Data collection: *APEX2* (Bruker, 2008 ▶); cell refinement: *SAINT* (Bruker, 2008 ▶); data reduction: *SAINT*; program(s) used to solve structure: *SHELXS97* (Sheldrick, 2008 ▶); program(s) used to refine structure: *SHELXL97* (Sheldrick, 2008 ▶); molecular graphics: CaRine (Boudias & Monceau, 1998 ▶) and *ORTEP*-3 for Windows (Farrugia, 1997 ▶); software used to prepare material for publication: *SHELXTL* (Sheldrick, 2008 ▶).

## Supplementary Material

Crystal structure: contains datablocks I, global. DOI: 10.1107/S1600536810026942/wm2370sup1.cif

Structure factors: contains datablocks I. DOI: 10.1107/S1600536810026942/wm2370Isup2.hkl

Additional supplementary materials:  crystallographic information; 3D view; checkCIF report

## Figures and Tables

**Table 1:** Selected bond lengths (Å)

P1—O6	1.4841 (8)
P1—O12	1.4850 (8)
P1—O5	1.5881 (7)
P1—O10	1.5887 (9)
P2—O11	1.4810 (9)
P2—O7^i^	1.4822 (8)
P2—O1	1.5926 (8)
P2—O2	1.5941 (8)
P3—O3^ii^	1.4806 (8)
P3—O4	1.4832 (8)
P3—O5	1.5902 (8)
P3—O1^iii^	1.5980 (8)
P4—O8^iv^	1.4777 (8)
P4—O9^v^	1.4829 (8)
P4—O2	1.5874 (8)
P4—O10	1.5973 (9)

Symmetry codes: (i) 
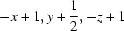
; (ii) 
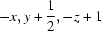
; (iii) 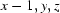; (iv) 
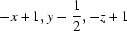
; (v) 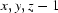.

**Fig. 1.**
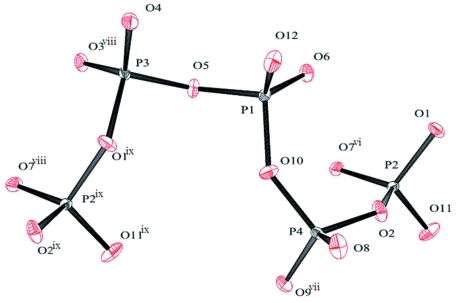
View of the repeating unit of the (PO3)∞ chains. Displacement ellipsoids are drawn at the 50% probability level. [Symmetry codes: (vi) -x + 1, y + 1/2, -z + 1; (vii) x, y, z - 1; (viii) -x, y + 1/2, -z + 1; (ix) x - 1, y, z.]

**Fig. 2.**
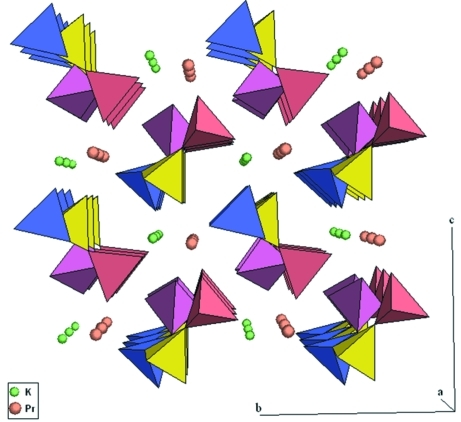
Projection along [100] showing the pseudo-tetragonal rod packing of the infinite (PO3) chains.

**Fig. 3.**
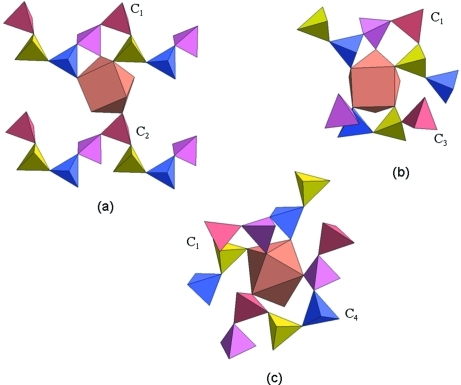
Details of the connections between the (PO3)∞ chains and the PrO8 square antiprisms: a) view showing the tridentate attachement of one chain (C1) and the connection of the second chain (C2) parallel and opposite to the first one. b) view showing the bidentate attachement of the third chain (C3) adjacent and orthogonal to the first one. c) view showing the bidentate attachement of the fourth chain (C4) also orthogonal and adjacent to the first one.

## supplementary crystallographic information

### Comment

Long chains polyphosphates with general formula *A*^I^*B*^III^(PO_3_)_4_ have been classified into seven structural types, labelled from I to VII (Durif, 1995; Jaoudi *et al.* 2003). All long-chains polyphosphates of formula K*Ln*(PO_3_)_4_ (*Ln* = rare earth elements) reported up to now (Zhu *et al.*, 2009; Horchani-Naifer *et al.*, 2008; Parreu *et al.*, 2006; Xing *et al.*, 1987; Ninghai *et al.*, 1984; Lin *et al*., 1983; Krutik *et al.*, 1980; Hong *et al.*, 1975) adopt type III except for KYb(PO_3_)_4_ (Palkina et *al.* 1981) which is the only presently known member of type V. Most of these potassium polyphosphates are dimorphic and crystallize with both the type III and the type IV polymorphs. KCe(PO_3_)_4_ which has been shown to crystallize with either the type II and the type III is the first exception. The second exception is concerned with the Er member of this series presenting the type VII polymorph besides both type III and type IV polymorphs. Moreover, type III long-chain polyphosphates do not exist for monovalent cations other than K^+^. The structure of the title compound also fits in this type III isotypic series.

The crystal structure of the title compound is built from crenelated chains with a repeating unit of four corner-sharing tetrahedra, as displayed in Fig. 1. The chains are further linked by isolated PrO_8_ square antiprisms to form the three-dimensional framework. Each PrO_8_ polyhedron (Pr—O distances range from 2.3787 (9) to 2.5091 (8) Å) is connected through vertices to four (PO_3_)_∞_ chains stacked in a pseudo-tetragonal rod packing as shown in Fig. 2. Figure 2 also shows that within this pseudo-tetragonal rod packing, two adjacent chains are twisted by *ca*. 90 ° whereas two opposite chains are parallel. The relative disposition of the chains running along the [100] direction accounts for the strong non-centrosymmetric character of the structure. Figure 3 displays details of the connections between the PrO_8_ square antiprisms and the four chains surrounding each antiprism. One of the four chains (labelled C_1_) is attached in a tridentate fashion on a triangular face of the square antiprism whereas the opposite and parallel chain (labelled C_2_) is connected only through a vertex (Fig. 3a). The two other chains which are adjacent to the first one are attached in a bidentate fashion. The first of these two chains (labelled C_3_) is linked through a bidentate diphosphate group attached on one side of one square face of the square antiprism (Fig. 3b). The second chain (labelled C_4_) is connected at the ends of one diagonal of the second square face of the antiprism (Fig. 3c) through corners of the terminal PO_4_ groups of the crenel-shaped tetraphosphate group corresponding to the repeating unit of the chain. This polyhedral linkage delimits channels running along [010] where the K^+^ions lie in a highly distorted environment defined by eight oxygen atoms at distances ranging from 2.7908 (9) to 3.1924 (11) Å.

For the cyclophosphate structure with the same composition KPr(PO_3_)_4_, see:  (Zhou *et al.*, 1987).

### Experimental

Crystals of the title compound were synthesized by reacting Pr_6_O_11_ with (NH_4_)H_2_PO_4_ and K_2_CO_3_ in a platinum crucible. A mixture of these reagents in the molar ratio 14: 66: 20 was used for the synthesis. The mixture has first been heated at 473 K for 12 h and then the temperature has been increased up to 573 K and maintained for 12 h before to be raised at 853 K and kept for 24 additional hours. At the end of this heating step, the muffle furnace was cooled down first to 673 K at the rate of 2 K h^-1^ and subsequently to room temperature by switching the power off. Single crystals were extracted from the batch by leaching with hot water.

### Refinement

The highest residual peak in the final difference Fourier map was located 0.46 Å from atom Pr and the deepest hole was located 0.47 Å from atom K.

### Crystal data

**Table:**

KPr(PO3)_4_	*F*(000) = 468
*M**_r_* = 495.89	*D*_x_ = 3.331 Mg m^−^^3^
Monoclinic, *P*2_1_	Mo *K*α radiation, λ = 0.71073 Å
Hall symbol: P 2yb	Cell parameters from 9960 reflections
*a* = 7.2872 (2) Å	θ = 2.8–57.3°
*b* = 8.4570 (3) Å	µ = 6.06 mm^−^^1^
*c* = 8.0268 (2) Å	*T* = 296 K
β = 91.994 (1)°	Prism, green
*V* = 494.37 (3) Å^3^	0.29 × 0.21 × 0.16 mm
*Z* = 2	

### Data collection

**Table:**

Bruker APEXII CCD diffractometer	13443 independent reflections
Radiation source: fine-focus sealed tube	13257 reflections with *I* > 2σ(*I*)
graphite	*R*_int_ = 0.034
Detector resolution: 8.3333 pixels mm^-1^	θ_max_ = 57.4°, θ_min_ = 3.7°
ω and φ scans	*h* = −17→17
Absorption correction: multi-scan (*SADABS*; Bruker, 2008)	*k* = −19→20
*T*_min_ = 0.448, *T*_max_ = 0.751	*l* = −18→18
45940 measured reflections	

### Refinement

**Table:**

Refinement on *F*^2^	Secondary atom site location: difference Fourier map
Least-squares matrix: full	*w* = 1/[σ^2^(*F*_o_^2^) + (0.*P*)^2^ + 0.0715*P*] where *P* = (*F*_o_^2^ + 2*F*_c_^2^)/3
*R*[*F*^2^ > 2σ(*F*^2^)] = 0.018	(Δ/σ)_max_ = 0.004
*wR*(*F*^2^) = 0.043	Δρ_max_ = 2.86 e Å^−^^3^
*S* = 1.07	Δρ_min_ = −2.41 e Å^−^^3^
13443 reflections	Extinction correction: *SHELXL97* (Sheldrick, 2008), Fc^*^=kFc[1+0.001xFc^2^λ^3^/sin(2θ)]^-1/4^
164 parameters	Extinction coefficient: 0.0305 (6)
1 restraint	Absolute structure: Flack (1983), 6278 Friedel pairs
0 constraints	Flack parameter: 0.022 (3)
Primary atom site location: structure-invariant direct methods	

### Special details

**Table:**

Geometry. All e.s.d.'s (except the e.s.d. in the dihedral angle between two l.s. planes) are estimated using the full covariance matrix. The cell e.s.d.'s are taken into account individually in the estimation of e.s.d.'s in distances, angles and torsion angles; correlations between e.s.d.'s in cell parameters are only used when they are defined by crystal symmetry. An approximate (isotropic) treatment of cell e.s.d.'s is used for estimating e.s.d.'s involving l.s. planes.
Refinement. Refinement of *F*^2^ against ALL reflections. The weighted *R*-factor *wR* and goodness of fit *S* are based on *F*^2^, conventional *R*-factors *R* are based on *F*, with *F* set to zero for negative *F*^2^. The threshold expression of *F*^2^ > σ(*F*^2^) is used only for calculating *R*-factors(gt) *etc*. and is not relevant to the choice of reflections for refinement. *R*-factors based on *F*^2^ are statistically about twice as large as those based on *F*, and *R*- factors based on ALL data will be even larger.

### Fractional atomic coordinates and isotropic or equivalent isotropic displacement parameters (Å^2^)

**Table:**

	*x*	*y*	*z*	*U*_iso_*/*U*_eq_	
K	0.77227 (5)	0.31500 (7)	0.72001 (4)	0.02909 (8)	
Pr	0.265282 (4)	0.119995 (8)	0.757982 (4)	0.00552 (1)	
P1	0.50137 (3)	0.26677 (3)	0.38019 (3)	0.00690 (3)	
P2	0.93476 (3)	0.24280 (3)	0.09865 (3)	0.00685 (3)	
P3	0.12252 (3)	0.37267 (3)	0.39531 (3)	0.00674 (3)	
P4	0.60346 (3)	0.04117 (3)	0.10444 (3)	0.00675 (3)	
O1	1.04301 (12)	0.23711 (10)	0.27436 (11)	0.01360 (11)	
O2	0.81354 (11)	0.08886 (10)	0.12702 (13)	0.01412 (12)	
O3	−0.03801 (11)	0.02642 (9)	0.65100 (11)	0.01191 (10)	
O4	0.11151 (11)	0.31458 (11)	0.56903 (9)	0.01188 (10)	
O5	0.33093 (10)	0.38001 (9)	0.34329 (10)	0.01061 (9)	
O6	0.66909 (10)	0.36646 (10)	0.38630 (10)	0.01127 (9)	
O7	0.18250 (12)	−0.11539 (10)	0.92653 (10)	0.01172 (10)	
O8	0.41427 (12)	0.38104 (10)	0.82424 (13)	0.01527 (12)	
O9	0.53826 (12)	0.07174 (11)	0.93036 (10)	0.01395 (12)	
O10	0.49263 (14)	0.16280 (13)	0.21526 (13)	0.01754 (15)	
O11	1.06463 (16)	0.21154 (13)	−0.03535 (13)	0.01866 (16)	
O12	0.46952 (13)	0.16652 (11)	0.52829 (11)	0.01411 (12)	

### Atomic displacement parameters (Å^2^)

**Table:**

	*U*^11^	*U*^22^	*U*^33^	*U*^12^	*U*^13^	*U*^23^
K	0.01717 (11)	0.0542 (3)	0.01579 (10)	0.00955 (13)	−0.00159 (8)	−0.00090 (12)
Pr	0.00546 (1)	0.00563 (1)	0.00548 (1)	−0.00032 (1)	0.00021 (1)	0.00006 (1)
P1	0.00666 (7)	0.00665 (7)	0.00750 (7)	0.00002 (5)	0.00174 (5)	0.00055 (5)
P2	0.00649 (6)	0.00695 (7)	0.00716 (7)	−0.00010 (5)	0.00069 (5)	−0.00144 (5)
P3	0.00623 (6)	0.00685 (7)	0.00704 (7)	0.00079 (5)	−0.00105 (5)	0.00031 (5)
P4	0.00704 (7)	0.00576 (6)	0.00735 (7)	−0.00051 (5)	−0.00141 (5)	−0.00019 (5)
O1	0.0159 (3)	0.0098 (2)	0.0145 (3)	0.0010 (2)	−0.0082 (2)	−0.00244 (18)
O2	0.0083 (2)	0.0108 (2)	0.0229 (3)	−0.00354 (17)	−0.0037 (2)	0.0027 (2)
O3	0.0102 (2)	0.0084 (2)	0.0168 (3)	−0.00284 (17)	−0.00320 (19)	−0.00037 (18)
O4	0.0114 (2)	0.0160 (3)	0.0082 (2)	0.0013 (2)	0.00038 (16)	0.00301 (18)
O5	0.00730 (18)	0.0111 (2)	0.0135 (2)	0.00173 (16)	0.00166 (16)	0.00354 (18)
O6	0.00779 (19)	0.0116 (2)	0.0145 (2)	−0.00191 (17)	0.00084 (16)	0.00167 (19)
O7	0.0141 (2)	0.0103 (2)	0.0106 (2)	−0.00433 (19)	−0.00116 (18)	0.00117 (17)
O8	0.0131 (3)	0.0090 (2)	0.0235 (4)	0.00183 (19)	−0.0015 (2)	−0.0067 (2)
O9	0.0143 (3)	0.0186 (3)	0.0087 (2)	0.0028 (2)	−0.00406 (18)	0.0001 (2)
O10	0.0157 (3)	0.0201 (3)	0.0169 (3)	0.0020 (3)	0.0016 (2)	−0.0106 (3)
O11	0.0206 (4)	0.0191 (3)	0.0171 (3)	0.0030 (3)	0.0121 (3)	−0.0029 (3)
O12	0.0141 (3)	0.0144 (3)	0.0142 (3)	0.0033 (2)	0.0059 (2)	0.0074 (2)

### Geometric parameters (Å, °)

**Table:**

K—O4^i^	2.7908 (9)	P2—O7^vi^	1.4822 (8)
K—O6	2.7909 (9)	P2—O1	1.5926 (8)
K—O8	2.8231 (10)	P2—O2	1.5941 (8)
K—O3^i^	2.8684 (10)	P2—K^vii^	3.2805 (4)
K—O7^ii^	2.9050 (9)	P3—O3^viii^	1.4806 (8)
K—O12	2.9285 (11)	P3—O4	1.4832 (8)
K—O11^iii^	2.9781 (13)	P3—O5	1.5902 (8)
K—O9	3.1924 (11)	P3—O1^ix^	1.5980 (8)
K—P2^iii^	3.2805 (4)	P4—O8^v^	1.4777 (8)
K—P1	3.3360 (4)	P4—O9^vii^	1.4829 (8)
Pr—O11^iv^	2.3787 (9)	P4—O2	1.5874 (8)
Pr—O9	2.4180 (8)	P4—O10	1.5973 (9)
Pr—O12	2.4414 (8)	O1—P3^i^	1.5980 (8)
Pr—O3	2.4731 (7)	O3—P3^x^	1.4806 (8)
Pr—O4	2.4791 (8)	O3—K^ix^	2.8684 (10)
Pr—O6^v^	2.4912 (8)	O4—K^ix^	2.7908 (9)
Pr—O7	2.4928 (8)	O6—Pr^vi^	2.4912 (8)
Pr—O8	2.5091 (8)	O7—P2^v^	1.4822 (8)
P1—O6	1.4841 (8)	O7—K^xi^	2.9051 (9)
P1—O12	1.4850 (8)	O8—P4^vi^	1.4776 (8)
P1—O5	1.5881 (7)	O9—P4^iii^	1.4829 (8)
P1—O10	1.5887 (9)	O11—Pr^xii^	2.3787 (9)
P2—O11	1.4810 (9)	O11—K^vii^	2.9782 (13)
			
O4^i^—K—O6	78.26 (2)	O12—Pr—O8	75.38 (3)
O4^i^—K—O8	166.08 (3)	O3—Pr—O8	136.89 (3)
O6—K—O8	91.87 (3)	O4—Pr—O8	74.26 (3)
O4^i^—K—O3^i^	58.32 (3)	O6^v^—Pr—O8	140.10 (3)
O6—K—O3^i^	93.61 (3)	O7—Pr—O8	134.32 (3)
O8—K—O3^i^	133.01 (3)	O11^iv^—Pr—K^ix^	48.64 (3)
O4^i^—K—O7^ii^	110.62 (3)	O9—Pr—K^ix^	147.29 (2)
O6—K—O7^ii^	157.42 (3)	O12—Pr—K^ix^	116.78 (2)
O8—K—O7^ii^	75.23 (3)	O3—Pr—K^ix^	46.23 (2)
O3^i^—K—O7^ii^	108.78 (3)	O4—Pr—K^ix^	44.44 (2)
O4^i^—K—O12	115.71 (3)	O6^v^—Pr—K^ix^	120.771 (19)
O6—K—O12	52.38 (2)	O7—Pr—K^ix^	97.96 (2)
O8—K—O12	63.48 (3)	O8—Pr—K^ix^	91.99 (2)
O3^i^—K—O12	83.86 (3)	O11^iv^—Pr—K	120.16 (3)
O7^ii^—K—O12	131.14 (3)	O9—Pr—K	51.68 (2)
O4^i^—K—O11^iii^	70.23 (2)	O12—Pr—K	45.47 (3)
O6—K—O11^iii^	147.28 (3)	O3—Pr—K	155.08 (2)
O8—K—O11^iii^	120.66 (3)	O4—Pr—K	94.44 (2)
O3^i^—K—O11^iii^	62.54 (3)	O6^v^—Pr—K	97.156 (19)
O7^ii^—K—O11^iii^	50.31 (2)	O7—Pr—K	127.02 (2)
O12—K—O11^iii^	136.81 (3)	O8—Pr—K	43.25 (2)
O4^i^—K—O9	136.58 (3)	K^ix^—Pr—K	130.558 (15)
O6—K—O9	118.49 (3)	O6—P1—O12	116.70 (5)
O8—K—O9	56.95 (3)	O6—P1—O5	107.54 (5)
O3^i^—K—O9	79.85 (3)	O12—P1—O5	110.52 (5)
O7^ii^—K—O9	70.04 (3)	O6—P1—O10	110.55 (5)
O12—K—O9	66.11 (2)	O12—P1—O10	110.38 (6)
O11^iii^—K—O9	81.08 (3)	O5—P1—O10	99.78 (5)
O4^i^—K—P2^iii^	96.054 (18)	O6—P1—K	55.97 (3)
O6—K—P2^iii^	174.10 (2)	O12—P1—K	61.30 (4)
O8—K—P2^iii^	94.00 (2)	O5—P1—K	121.03 (3)
O3^i^—K—P2^iii^	81.93 (2)	O10—P1—K	138.98 (4)
O7^ii^—K—P2^iii^	26.858 (16)	O11—P2—O7^vi^	115.17 (6)
O12—K—P2^iii^	130.40 (2)	O11—P2—O1	109.17 (6)
O11^iii^—K—P2^iii^	26.821 (17)	O7^vi^—P2—O1	114.25 (5)
O9—K—P2^iii^	64.676 (17)	O11—P2—O2	109.22 (6)
O4^i^—K—P1	98.784 (19)	O7^vi^—P2—O2	111.14 (5)
O6—K—P1	26.147 (16)	O1—P2—O2	96.24 (5)
O8—K—P1	74.92 (2)	O11—P2—K^vii^	65.14 (5)
O3^i^—K—P1	90.78 (2)	O7^vi^—P2—K^vii^	62.31 (3)
O7^ii^—K—P1	150.12 (2)	O1—P2—K^vii^	167.90 (4)
O12—K—P1	26.409 (16)	O2—P2—K^vii^	95.76 (4)
O11^iii^—K—P1	153.20 (3)	O3^viii^—P3—O4	119.42 (5)
O9—K—P1	92.400 (19)	O3^viii^—P3—O5	107.01 (5)
P2^iii^—K—P1	156.78 (2)	O4—P3—O5	110.07 (4)
O4^i^—K—P4^vi^	148.57 (3)	O3^viii^—P3—O1^ix^	109.79 (4)
O6—K—P4^vi^	96.19 (2)	O4—P3—O1^ix^	107.65 (5)
O8—K—P4^vi^	21.809 (17)	O5—P3—O1^ix^	101.41 (5)
O3^i^—K—P4^vi^	152.95 (2)	O3^viii^—P3—K^ix^	90.09 (4)
O7^ii^—K—P4^vi^	65.122 (18)	O5—P3—K^ix^	150.30 (3)
O12—K—P4^vi^	82.33 (2)	O1^ix^—P3—K^ix^	95.04 (4)
O11^iii^—K—P4^vi^	115.28 (2)	O8^v^—P4—O9^vii^	119.69 (6)
O9—K—P4^vi^	73.308 (19)	O8^v^—P4—O2	106.59 (5)
P2^iii^—K—P4^vi^	89.502 (11)	O9^vii^—P4—O2	109.84 (5)
P1—K—P4^vi^	87.099 (10)	O8^v^—P4—O10	108.77 (6)
O4^i^—K—P3^i^	20.271 (16)	O9^vii^—P4—O10	105.12 (6)
O6—K—P3^i^	58.589 (17)	O2—P4—O10	106.09 (5)
O8—K—P3^i^	147.16 (3)	O8^v^—P4—K^v^	45.22 (4)
O3^i^—K—P3^i^	68.374 (19)	O9^vii^—P4—K^v^	103.55 (4)
O7^ii^—K—P3^i^	126.64 (2)	O2—P4—K^v^	145.07 (4)
O12—K—P3^i^	102.092 (19)	O10—P4—K^v^	73.90 (4)
O11^iii^—K—P3^i^	90.45 (2)	P2—O1—P3^i^	132.40 (6)
O9—K—P3^i^	147.36 (2)	P4—O2—P2	136.74 (6)
P2^iii^—K—P3^i^	115.882 (11)	P3^x^—O3—Pr	136.64 (5)
P1—K—P3^i^	81.085 (10)	P3^x^—O3—K^ix^	126.63 (4)
P4^vi^—K—P3^i^	137.479 (17)	Pr—O3—K^ix^	95.26 (3)
O11^iv^—Pr—O9	99.75 (4)	P3—O4—Pr	139.19 (5)
O11^iv^—Pr—O12	151.63 (3)	P3—O4—K^ix^	119.05 (4)
O9—Pr—O12	87.04 (3)	Pr—O4—K^ix^	97.10 (3)
O11^iv^—Pr—O3	77.45 (4)	P1—O5—P3	132.48 (5)
O9—Pr—O3	148.45 (3)	P1—O6—Pr^vi^	130.14 (5)
O12—Pr—O3	110.50 (3)	P1—O6—K	97.89 (4)
O11^iv^—Pr—O4	86.26 (3)	Pr^vi^—O6—K	122.05 (3)
O9—Pr—O4	143.85 (3)	P2^v^—O7—Pr	136.16 (5)
O12—Pr—O4	72.87 (3)	P2^v^—O7—K^xi^	90.83 (4)
O3—Pr—O4	67.70 (3)	Pr—O7—K^xi^	132.10 (3)
O11^iv^—Pr—O6^v^	137.47 (3)	P4^vi^—O8—Pr	147.72 (6)
O9—Pr—O6^v^	87.33 (3)	P4^vi^—O8—K	112.97 (5)
O12—Pr—O6^v^	69.86 (3)	Pr—O8—K	99.24 (3)
O3—Pr—O6^v^	75.34 (3)	P4^iii^—O9—Pr	143.02 (6)
O4—Pr—O6^v^	112.14 (3)	P4^iii^—O9—K	116.82 (5)
O11^iv^—Pr—O7	73.45 (4)	Pr—O9—K	91.87 (3)
O9—Pr—O7	76.38 (3)	P1—O10—P4	144.49 (7)
O12—Pr—O7	134.75 (3)	P2—O11—Pr^xii^	171.29 (7)
O3—Pr—O7	72.70 (3)	P2—O11—K^vii^	88.04 (5)
O4—Pr—O7	138.54 (3)	Pr^xii^—O11—K^vii^	94.52 (4)
O6^v^—Pr—O7	67.60 (3)	P1—O12—Pr	144.39 (5)
O11^iv^—Pr—O8	80.66 (4)	P1—O12—K	92.29 (5)
O9—Pr—O8	71.69 (3)	Pr—O12—K	98.06 (3)

Symmetry codes: (i) *x*+1, *y*, *z*; (ii) −*x*+1, *y*+1/2, −*z*+2; (iii) *x*, *y*, *z*+1; (iv) *x*−1, *y*, *z*+1; (v) −*x*+1, *y*−1/2, −*z*+1; (vi) −*x*+1, *y*+1/2, −*z*+1; (vii) *x*, *y*, *z*−1; (viii) −*x*, *y*+1/2, −*z*+1; (ix) *x*−1, *y*, *z*; (x) −*x*, *y*−1/2, −*z*+1; (xi) −*x*+1, *y*−1/2, −*z*+2; (xii) *x*+1, *y*, *z*−1.
